# Successful rechallenge with cetuximab after progression with nivolumab for recurrent cervical lymph node metastasis from carcinoma of the tongue

**DOI:** 10.1002/ccr3.4464

**Published:** 2021-07-16

**Authors:** Yasuyuki Asada, Chitoshi Teramura, Takuma Wada, Yoshisato Machida, Shinya Koshinuma, Gaku Yamamoto

**Affiliations:** ^1^ Department of Oral and Maxillofacial Surgery Shiga University of Medical Science Shiga Japan; ^2^ Department of Oral and Maxillofacial Surgery Otsu City Hospital Shiga Japan

**Keywords:** carcinoma of the tongue, cetuximab, metastasis, nivolumab, rechallenge

## Abstract

We can infer that the immunostimulatory effect of nivolumab and reactivation of cetuximab enhance the antitumor effect of the therapy.

## Graphical abstract

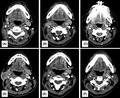

## 1 INTRODUCTION

We demonstrate the effectiveness of readministering cetuximab as a salvage chemotherapeutic agent after nivolumab administration to a patient with a recurrence of cervical lymph node metastasis after tongue cancer surgery. We can infer that the immunostimulatory effect of nivolumab and reactivation of cetuximab enhance the antitumor effect of the therapy.

In March 2017, nivolumab, an immune checkpoint inhibitor, was approved for treating squamous cell carcinoma of the head and neck with recurrence or metastasis in Japan. Nivolumab differs from conventional chemotherapeutic drugs; it is known to elicit a reaction and has been reported to be highly effective for lung cancer when administered after immunotherapy. However, few reports of its use for head and neck cancer treatment are available. Here, we present the case of a patient who had recurrent cervical lymph node metastasis due to tongue cancer. The administration of cetuximab and nivolumab led to increased metastasis; however, the readministration of cetuximab resulted in a remarkable response.

## 2 CASE REPORT

**FIGURE 1:**
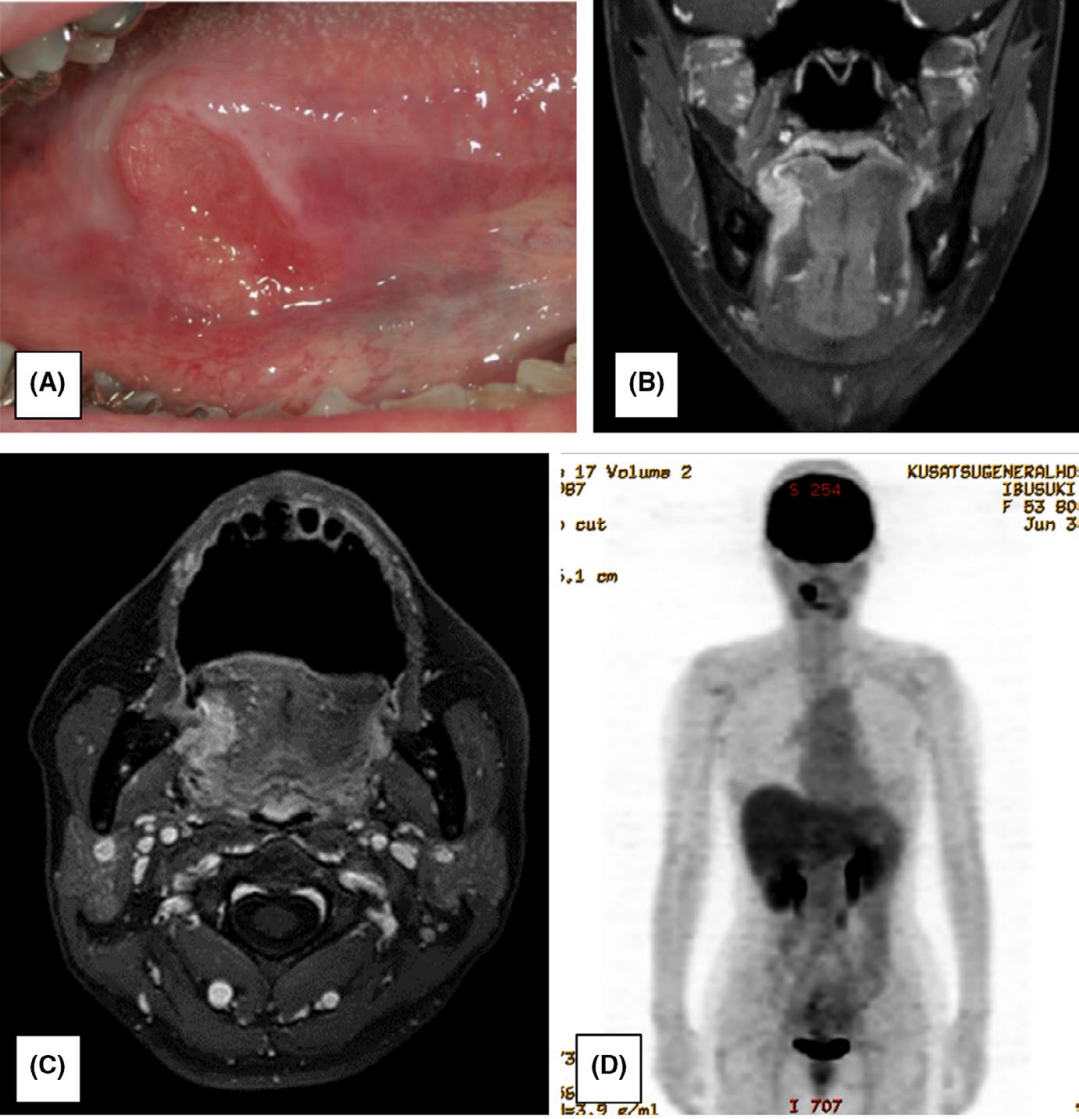
(A) Photograph taken during the patient's initial visit, showing an ulcerative lesion measuring approximately 22 mm in diameter on the right tongue margin. (B) Magnetic resonance image showing frontal disconnection. (C) Magnetic resonance image in the horizontal view. An invasive lesion showing a contrast effect with gadolinium was observed on the right tongue margin. (D) Positron emission tomography image showing a high accumulation of fluorodeoxyglucose in the right tongue margin

A 55‐year‐old patient (height, 160 cm; weight, 52 kg; with good nutritional status) presented to our hospital with an ulcer on the right tongue margin in June 2015. The patient had been previously diagnosed with endometrial atypical growth by the obstetrics and gynecology department of a nearby hospital in December 2012. The patient neither consumed alcohol nor smoked and had no family history of cancer. An extraoral examination revealed no abnormal findings in the cervical lymph nodes; however, an intraoral examination using magnetic resonance imaging (MRI) showed an ulcerative lesion with a diameter of approximately 22 mm on the right tongue margin (Figure 1A). Moreover, infiltrative lesions showing a contrast effect with gadolinium were observed in the right tongue area (Figure 1B,C). Positron emission tomography (PET) showed a high accumulation of fluorodeoxyglucose in the right tongue margin, but not in other organs (Figure 1D). Based on these findings and the pathological evaluation of an intraoral biopsy specimen, we diagnosed the patient with stage II (T2N0M0) squamous cell carcinoma of the tongue.^1^

**FIGURE 2:**
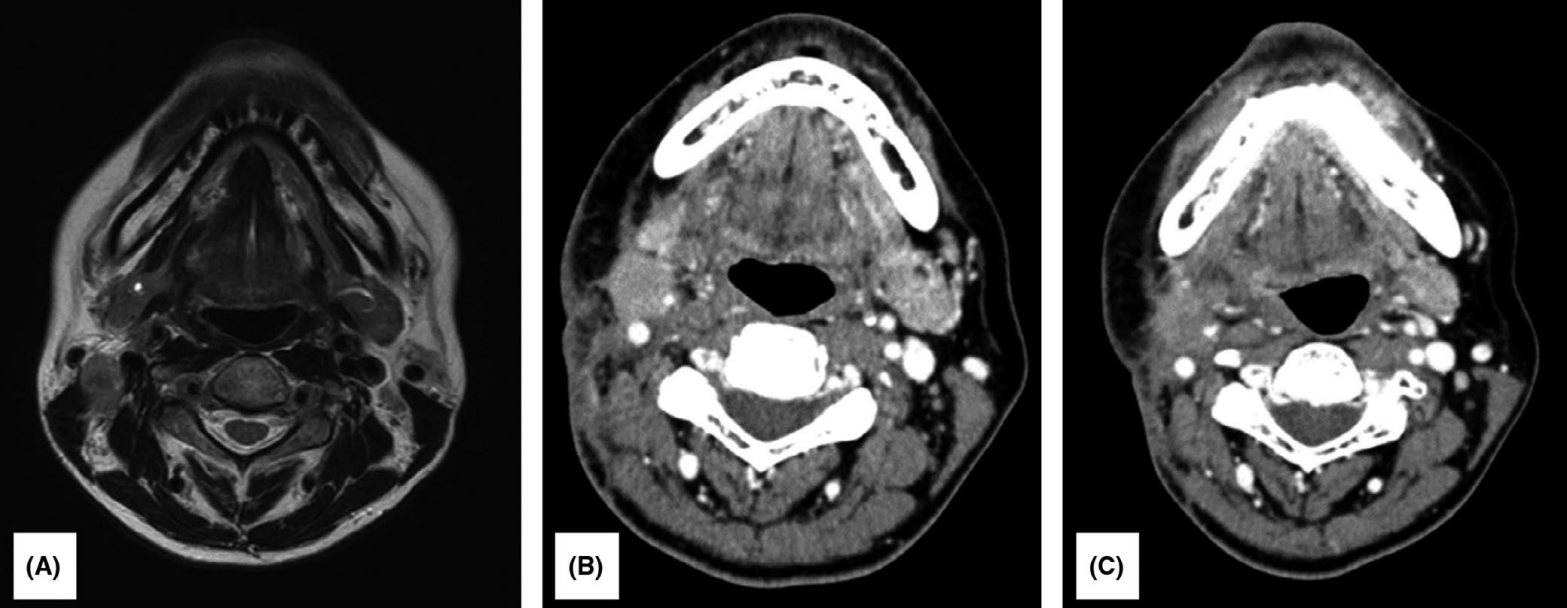
(A) Magnetic resonance image taken 7 months after tongue cancer surgery. The right superior‐internal jugular vein lymph node had become swollen with a minor axis of approximately 12 mm, and the T2‐weighted image shows a region with moderate signal intensity that included a part of the low‐signal region. (B) Computed tomography image taken 3 months after right neck dissection. The right submandibular lymph node had become swollen with a minor axis of approximately 15 mm, and the boundary with the surrounding area was unclear. (C) Computed tomography image taken after chemoradiotherapy. No change in the size of the right submandibular lymph node was observed, and the boundary with the surrounding area was unclear

In July 2015, the patient underwent partial right tongue resection under general anesthesia. Postoperative adjuvant therapy was not administered because the surgical margin was negative. Although recurrence was not observed locally or in the neck, swelling of the right cervical region was observed in December 2015 and the patient was followed up with monthly MRI thereafter. MRI performed in February 2016 showed that the right superior‐internal jugular vein lymph node had a minor axis of approximately 12 mm. The patient was then diagnosed with late cervical lymph node metastasis of tongue cancer because MRI showed a region with moderate signal intensity that included a part of the low‐signal region (Figure 2A). In the same month, the patient underwent right total neck dissection under general anesthesia. Postoperative adjuvant therapy was not administered at this time because histopathological diagnosis did not detect extracapsular invasion in the right superior‐internal jugular vein lymph node.

The patient was followed up, and swelling was observed in the lower part of the patient's right jaw in May 2016. Computed tomography (CT) showed that the right submandibular lymph node had become swollen with a minor axis of approximately 15 mm and that the boundary with the surrounding area was unclear (Figure 2B). Recurrence was confirmed after the patient was diagnosed with late cervical lymph node metastasis of tongue cancer, and chemoradiotherapy (cisplatin [cis‐diamminedichloroplatinum II] total dose, 300 mg/m^2^; radiotherapy total dose, 66 Gy) was initiated from the same month onward, which resulted in a stable disease (Figure 2C).

**FIGURE 3:**
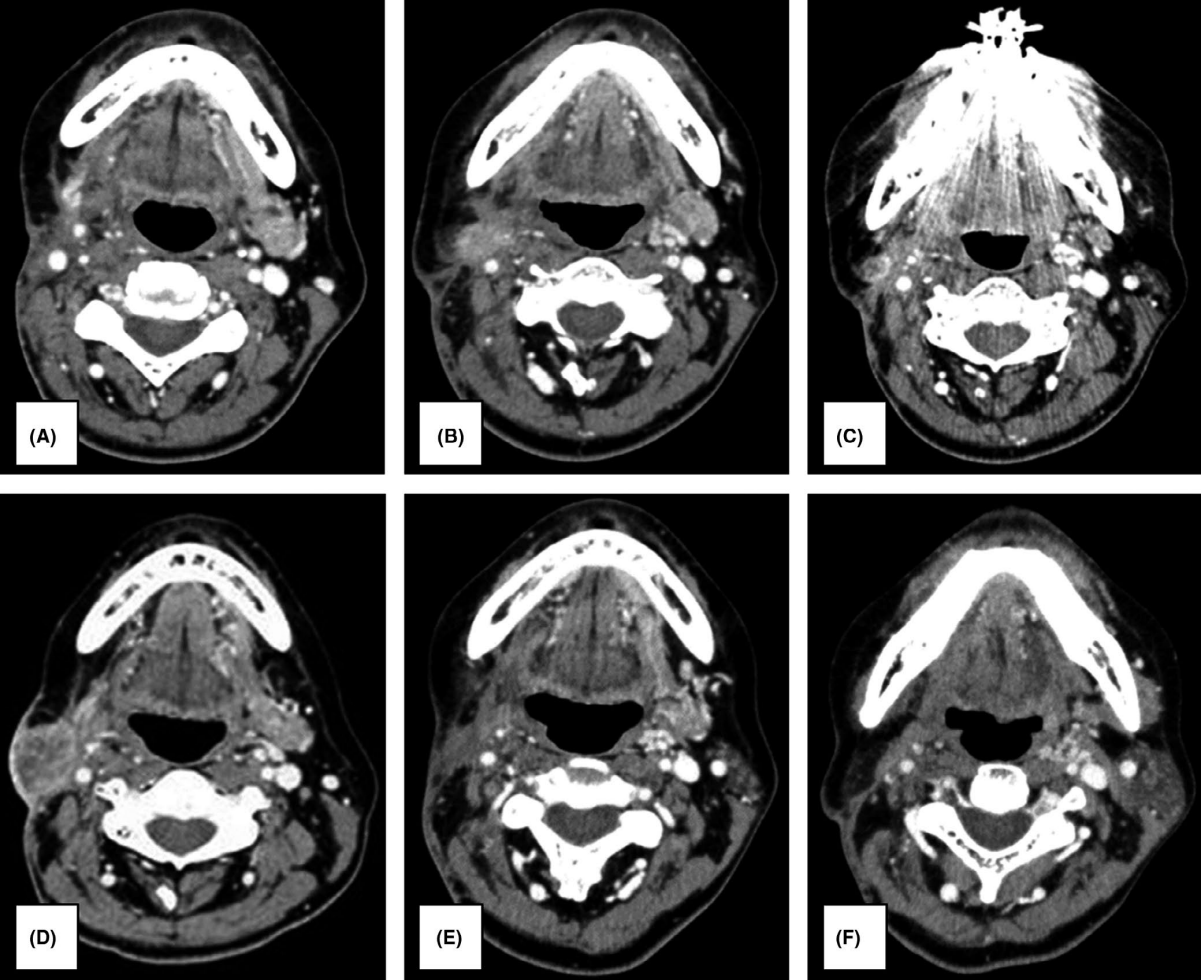
(A) Computed tomography image taken after three courses of cetuximab combination chemotherapy. The right submandibular recurrent lymph node had disappeared. (B) Computed tomography image taken 3 months after the maintenance therapy of cetuximab alone was initiated. Swelling of the right submandibular lymph node was observed. (C) Computed tomography image taken after six doses of nivolumab. Shrinkage of the right submandibular lymph node was observed. (D) Computed tomography image taken after 14 doses of nivolumab. Significant enlargement of the right submandibular lymph node was observed. (E) Computed tomography image taken after three courses of chemotherapy with cetuximab. Significant reduction of the right submandibular recurrent lymph node was observed. (F) Computed tomography image taken after 52 doses of cetuximab alone. The right submandibular lymph node had disappeared

Chemotherapy with cetuximab was initiated in September 2016. Cetuximab was initially administered at a dose of 400 mg/m^2^ and subsequently at a dose of 250 mg/m^2^ once weekly. Moreover, cisplatin was administered at a dose of 80 mg/m^2^ on Day 1, and fluorouracil was administered at a dose of 800 mg/m^2^ on Days 1– 4. CT was performed in December 2016, at the end of the three courses of cetuximab combination chemotherapy. The recurrent lymph node had disappeared and a complete response (CR) was therefore recorded (Figure 3A). Paronychia of the limbs, which was considered a skin reaction triggered by cetuximab, was observed; however, it only caused mild pain and improved with the application of a moisturizer. Cetuximab alone was maintained from the same month onward at a dose of 250 mg/m^2^ once weekly.

Another CT was performed in March 2017, 3 months after the maintenance therapy of cetuximab alone was initiated, which revealed that the right submandibular lymph node was swollen. Therefore, the treatment response was classified as progressive (Figure 3B). Nivolumab therapy was then initiated at a dose of 3 mg/kg once every 2 weeks. No immune‐related adverse events due to nivolumab were observed. CT was performed again in July 2017, following the administration of six doses of nivolumab, which revealed shrinkage of the right submandibular lymph node (Figure 3C). The treatment response was categorized as partial, and nivolumab administration was continued.

**FIGURE 4:**
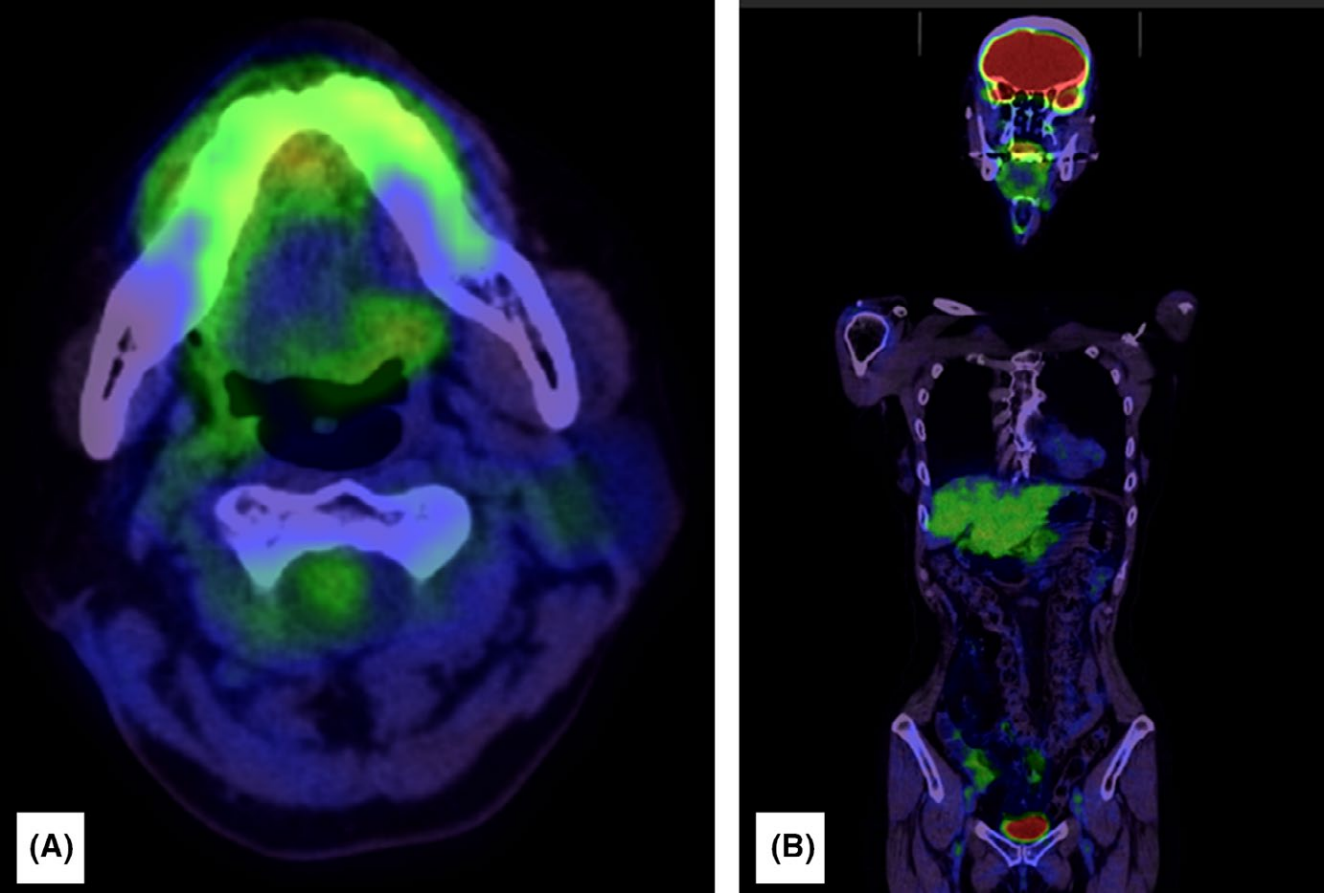
(A, B) Positron emission tomography images taken 20 months after complete response was achieved. No recurrence or metastasis was observed

In October 2017, however, after 14 courses of nivolumab, swelling of the right submandibular gland was observed; CT revealed a marked increase in the right submandibular lymph node size (Figure 3D). Thus, the patient was considered to have a progressive disease, and cetuximab combination chemotherapy was recontinued from the same month onward. The dose and dosing interval were the same as previously implemented. The next CT was performed in February 2018, following three courses with the readministration of cetuximab combination chemotherapy, which showed that the right submandibular recurrent lymph node was markedly reduced in size (Figure 3E). From the same month onward, the patient received a maintenance therapy of cetuximab alone. The final CT was performed in March 2019, after 52 doses of cetuximab. The submandibular lymph node had disappeared, and CR was achieved (Figure 3F). Cetuximab alone was still being administered as the maintenance therapy 20 months later. PET did not detect any recurrence or metastasis thereafter, and the course has been uneventful (Figure 4A,B).

## 3 DISCUSSION

With recent advances in drug therapies, such as molecular‐targeted therapies and immune checkpoint inhibitors, the role of drug therapy in the treatment of head and neck cancer is rapidly expanding and its scope is becoming more complex.^2^ In Japan, the anti‐epidermal growth factor receptor antibody cetuximab was approved as a molecular‐targeted drug for head and neck cancer in December 2012. Moreover, a 2017 subanalysis of the CheckMate 141 trial found that the anti‐programmed death 1 (PD‐1) antibody nivolumab had favorable effects in the treatment of recurrent and metastatic squamous cell carcinoma of the head and neck.^3^ Nivolumab is now being used as one of the standard treatments for head and neck cancer.^3^

Nivolumab has been shown to have responses and therapeutic effects that are distinct from those of conventional anticancer and molecular‐targeted drugs. Nivolumab treatment has been reported to have long‐lasting effects in successful cases and suppress the exacerbation of tumors for long periods in unchanged ones.^4^ Studies have suggested that salvage chemotherapy after the administration of previously approved immune checkpoint inhibitors is highly effective for treating non–small‐cell lung cancer.^5^, ^6^, ^7^

In the present study, recurrent lymph nodes disappeared after chemotherapy with cetuximab, but increased after the treatment was switched to cetuximab alone. Nivolumab was subsequently administered; however, because recurrent lymph nodes continued to increase, chemotherapy with cetuximab was initiated again. The patient subsequently showed a marked reduction in recurrent lymph nodes and continues to be in CR at present. These outcomes may be related to the reactivation of cetuximab and the immunostimulatory effect of nivolumab.

Regarding the reactivation of cetuximab, Santini et al.^8^ found tumor growth after cetuximab was administered and readministered as an adjuvant therapy in 21 of 39 patients with colorectal cancer, 53.8% of whom reported a response. Moreover, regarding the mechanism by which the readministration of cetuximab is effective, Aparicio and Caldas^9^ reported that there was a heterogeneous presence of cells that were sensitive to cetuximab and low‐signal cells were present in the tumor. They found that the administration of cetuximab (1) initially led to a reduction in the number of sensitive cells, an increase in the number of insensitive cells, and tumor growth, but (2) subsequently resulted in an increase in the number of sensitive cells. These findings suggest that the readministration of cetuximab shrinks tumors.

Regarding its immunostimulatory effect, nivolumab is a human immunoglobulin G4 anti‐PD‐1 antibody.^10^ PD‐1, a receptor from the CD28 family,^10^ is expressed in immune cells, such as differentiated effector T cells and B cells, after activation. The binding of antigen‐presenting cells to PD‐L1 and PD‐L2 ligands on lymphocytes, PD‐L1 expressed on tumor cells, and PD‐1 expressed on T cells, which transmits a negative signal to T cells, suppresses their activation.^10^

Nivolumab restores anticancer immune response by inhibiting the binding of PD‐1 to PD‐L1 and PD‐L2 and subsequently diminishing inhibitory signals to T cells.^10^ In addition, research has considered that conventional chemotherapy suppresses cancer cell growth and cytotoxicity.^4^ However, in recent years, it has become clear that immune cells represented by T cells are involved in long‐term clinical effects.^4^ Chemotherapeutic agents have been shown to act directly on immunocompetent and immunosuppressive cells as well as cancer cells, thereby affecting their antitumor effects.^4^

Cisplatin has been reported to increase the expression of tumor‐specific antigens and promote the induction of antigen‐associated cytotoxic T cells.^11^ In addition, paclitaxel is known to improve not only antigenicity by enhancing the expression of major histocompatibility complex class I molecules in cancer cells but also the ability of dendritic cells to present antigens to T cells by acting directly on the dendritic cells.^12^ Furthermore, fluorouracil has been shown to selectively induce apoptosis in myeloid‐derived suppressor cells and increase their antitumor effect.^13^

Change in the cancer immune microenvironment due to the administration of nivolumab influences the effects of succeeding chemotherapeutic agents used on immunocompetent and immunosuppressive cells, with the said influence being enhanced by its interaction with the original cell‐killing effect. Limited data on the successful cases of rescue chemotherapy after the administration of such immune checkpoint inhibitors in oral cancer highlight the need for more cases to be reported.

## 4 CONCLUSION

Our case demonstrated the effectiveness of readministering cetuximab as a salvage chemotherapeutic agent after administering nivolumab in a patient diagnosed with progressive disease for the recurrence of cervical lymph node metastasis after tongue cancer surgery. Our findings suggest that the immunostimulatory effect of nivolumab and reactivation of cetuximab enhance the antitumor effect of the therapy, which highlight the need to accumulate similar cases to further validate the findings.

## CONFLICTS OF INTERESTS

The authors declare that they have no competing interests.

## AUTHOR CONTRIBUTION

Yasuyuki Asada and Chitoshi Teramura: conception and design of study, acquisition of data, analysis and/or interpretation of data, and drafting the manuscript. Takuma Wada: acquisition of data. Yoshisato Machida: analysis and/or interpretation of data. Shinya Koshinuma: revising the manuscript critically for important intellectual content. Gaku Yamamoto: conception and design of study, revising the manuscript critically for important intellectual content. All authors critically revised the report, commented on drafts of the manuscript, and approved the final report.

## ETHICAL APPROVAL

Written informed consent was obtained from the patient to publish this case report, laboratory data, and accompanying clinical images. Any investigation on the patient was performed in accordance with the Declaration of Helsinki.

## Data Availability

The data that support the findings of this study are available from the corresponding author upon request.
